# Correlation between Human Papillomavirus Codetection Profiles and Cervical Intraepithelial Neoplasia in Japanese Women

**DOI:** 10.3390/microorganisms8121863

**Published:** 2020-11-25

**Authors:** Kaori Okayama, Hirokazu Kimura, Koji Teruya, Yasuyoshi Ishii, Kiyotaka Fujita, Masahiko Fujii, Mizue Oda, Toshiyuki Sasagawa, Mitsuaki Okodo

**Affiliations:** 1 Department of Medical Technology, Faculty of Health Sciences, Gunma Paz University, 1-7-1 Tonyamachi, Takasaki-shi, Gunma 370-0006, Japan; okayaman0811@std.kyorin-u.ac.jp (K.O.); h-kimura@paz.ac.jp (H.K.); fujita@paz.ac.jp (K.F.); 2 Department of Health and Welfare, Faculty of Health Sciences, Kyorin University, 5-4-1 Shimorenjaku, Mitaka-shi, Tokyo 181-8621, Japan; teruya@ks.kyorin-u.ac.jp; 3 Genki Plaza Medical Center for Health Care, 3-6-5 Iidabashi, Chiyoda-ku, Tokyo 102-0072, Japan; y-ishii@genkiplaza.or.jp (Y.I.); m-oda@genkiplaza.or.jp (M.O.); 4 Department of Medical Technology, Faculty of Health Sciences, Kyorin University, 5-4-1 Shimorenjaku, Mitaka-shi, Tokyo 181-8621, Japan; fujiim1951-1011@tbz.t-com.ne.jp; 5 Department of Obstetrics and Gynecology, Kanazawa Medical University, 1-1 Uchinadadaigaku, Kahoku-gun, Ishikawa 920-0293, Japan; tsasa@kanazawa-med.ac.jp

**Keywords:** human papillomavirus (HPV), cervical intraepithelial neoplasia (CIN), invasive cervical cancer (ICC), uniplex E6/E7 PCR method, high-risk human papillomavirus (HPV), codetection

## Abstract

Human papillomavirus (HPV) infection is thought to be strongly associated with the precarcinomatous state cervical intraepithelial neoplasia (CIN) and cervical carcinoma. To accurately assess the correlation between HPV detection profiles and CIN, the uniplex E6/E7 polymerase chain reaction (PCR) method was used. We detected HPV (37 genotypes) in 267 CIN cases. The detection of a single high-risk HPV genotype occurred in 69.7% of CIN1 and worse than CIN1 (CIN1+) cases whereas other types were detected in 11.6% of cases. Codetection of high-risk HPV genotypes occurred in 4.9% of CIN1+ cases. The high-risk genotype HPV16 was the most frequently detected genotype in CIN1+ lesions; the genotype HPV34 (not a high-risk type) was detected in some CIN3 cases. Furthermore, HPV codetection may not be associated with CIN grades. These results suggest that various HPV genotypes are associated with CIN across all analyzed cases.

## 1. Introduction

Human papillomavirus (HPV) is associated with various oncologic diseases, including benign tumors and carcinomas [1,2]. HPV may cause persistent infections, mostly in epithelial cells; it can also induce tumorigenic transformation [3,4]. During the transformation process, precarcinomatous states such as cervical intraepithelial neoplasia (CIN) may be present in the uterus [5,6]. These have the potential to develop into invasive cervical cancer (ICC) [7]. Therefore, long-term observation of CIN is important for the early diagnosis and treatment of ICC.

Currently, HPV is phylogenetically classified into over 100 genotypes [8]. Some, such as genotypes HPV16, 18, and 31, are more frequently detected in ICC, and considered high-risk [9]. Other genotypes, such as HPV6 and 11, are considered low-risk types [10]. Previous reports suggest that HPV coinfection occurs in CIN/ICC cells [11]. Various HPV genotypes have been detected in CIN/ICC cells, although the exact coinfection status in cells is unknown [12,13,14].

Some previous studies have reported CIN/ICC cells in which no HPV could be detected [15,16]. This may be a result of limitations with respect to detectable HPV genotypes [17,18]. For example, Onuki et al. [15] focused on the detection of high-risk HPV types in CIN/ICC cells. The subject of this report was exfoliated with cervical cells and may not reflect lesions due to sampling errors. Sampling errors during cervical swabs may be another source of negative HPV results [19]. Considering these findings, a new, comprehensive HPV detection method called “uniplex E6/E7 polymerase chain reaction (PCR)” has been developed, which is able to identify 39 different HPV genotypes [20]. The aim of this study was to investigate the HPV infection status of various genotypes as well as high-risk types in CIN1 and worse than CIN1 (CIN1+) lesions. This study may help to provide a model for reconsidering the genotype to be detected when introducing primary HPV screening for cervical cancer with HPV test in Japan.

## 2. Materials and Methods

### 2.1. Clinical Samples

Specimens were obtained from colposcopy-directed punch biopsies collected from 398 patients at the Genki Plaza Medical Center for Health Care, Tokyo, Japan, between 2014 and 2018. Colposcopy-directed punch biopsies contained cervical lesions (except for condyloma) from women with abnormal cytology findings. Cervical tissue biopsies were performed if subjects showed abnormal findings on cervical cytology after medical examination. Biopsy specimens were fixed in neutral buffered formalin and embedded in paraffin. Serial sections were cut from each formalin-fixed paraffin-embedded (FFPE) tissue block. The first section was stained with hematoxylin-eosin for histopathological evaluation; additional sections were used for the preparation of DNA and HPV genotyping. Biopsies were grouped according to morphological diagnosis. All case diagnoses were reviewed and reconfirmed by one pathologist. The mean age of the patients was 39.1 years (range, 19–67 years). No data regarding HPV vaccination history was collected from patients.

DNA isolation from CIN1+ FFPE tissue was performed using the hot sodium hydroxide and Tris (HotSHOT) method [21]. Briefly, a 4-μm thick section from the block was placed on a slide, deparaffinized in xylene, and treated with 100% ethanol to remove the xylene. The epithelium alone was scraped from the tissue on the slide using a needle. The epithelial tissues were lysed with 50 μL alkaline lysis solution (25 mM NaOH and 0.2 mM ethylenediaminetetraacetic acid [EDTA]; pH, 12.0) for 30 min at 95 °C. Tissues were then centrifuged at 13,200 rpm for 1 min and used directly as a DNA template. Human β-actin expression, determined using a PCR method, was used as an internal standard. HPV genotyping was performed for CIN1+ cases that tested positive for β-globin, used to demonstrate that amplifiable quality DNA was extracted from the specimens.

### 2.2. Ethical Approval

Samples were collected after written informed consent was obtained from subjects. The study protocol was approved by the Ethics Committee on Human Research of Kyorin University (H28–27). The protocol was implemented in accordance with approved guidelines.

### 2.3. HPV Genotyping

A new HPV-typing assay method, uniplex E6/E7 PCR, previously established by Okodo et al. [20] was used. The PCR reaction mixture included 1 × AmpliTaq Gold^®^ 360 buffer, 2 mM MgCl_2_, 0.025 U/µL AmpliTaq Gold 360 DNA Polymerase (Applied Biosystems, Foster City, CA, USA), 1 μL DNA, and 0.5 pM primers in a total volume of 25 μL. PCR amplification was performed using a thermal cycler, with 35 cycles of denaturation at 95 °C (30 s), annealing at 60 °C (30 s), and extension at 72 °C (30 s), including an initial denaturation step of 10 min and a final extension step of 5 min. As the PCR method occasionally provides false-positive results, each PCR experiment included DNase-free water as a negative control to eliminate the possibility of DNA contamination. HPV types were classified into two groups in the present study: high-risk genotypes [22] (HPV-16, -18, -31, -33, -35, -39, -45, -51, -52, -56, -58, and -59) and other genotypes (HPV-6, -11, -26, -30, -34, -40, -42, -44, -53, -54, -55, -61, -62, -66, -67, -68, -69, -70, -71, -73, -74, -81, -82, -84, -85, -89, and -90). The latter group included low-risk, probable high-risk, and undetermined risk types. Formalin fixation can cause extensive DNA damage, including cross-linking and fragmentation, which may affect HPV genotyping from FFPE [23]. Thus, some amplicons detected from FFPE tissues were validated using sequencing and BLAST analyses. Amplicons were purified and sequenced on an ABI 3730XL sequencer (Applied Biosystems, Foster City, CA, USA) using ABI BigDye™ Terminator v3.1 Cycle Sequencing Kits with AmpliTaq DNA polymerase (Applied Biosystems, Foster City, CA, USA) to confirm the target HPV type. Sequence data for each HPV type were obtained from the GenBank database using BLAST.

### 2.4. Statistical Analysis

Statistical analyses were performed using SPSS version 25.0 (IBM SPSS, Chicago, IL). Differences between groups were examined using residual analysis, based on results of Chi-squared test and one-way analysis of variance and according to the characteristics of the data distribution. In all cases, a *P*-value of < 0.05 was considered statistically significant.

## 3. Results

### 3.1. Correlation between Pathological Findings and HPV Detection Rates

Pathological examination suggested that 75.1% (299/398) of cases were CIN positive. These were further classified as 132 CIN1 lesions, 82 CIN2 lesions, and 85 CIN3 and worse than CIN3 (CIN3+) lesions (77 CIN3, 7 micro-invasive squamous carcinomas, and 1 invasive squamous carcinoma). Ninety-nine cases were benign lesions (9 atypical metaplasia and 90 chronic cervicitis). Mean ages for the CIN1, CIN2, and CIN3+ groups were 36.6 years (range, 19–60 years), 39.2 years (range, 20–61 years), and 40.4 years (range, 23–67 years), respectively. Patients with CIN1 were significantly younger than those with CIN2 (*p* = 0.049) and CIN3 (*p* = 0.007). In 89.3% (267/299) of CIN1+ tissues, amplifiable quality DNA was obtained based on β-globin positive results. Uniplex E6/E7 PCR detected evidence of HPV in 100% of CIN1+ samples. In total, of 39 possible HPV genotypes, 37 genotypes were detected, with only HPV26 and HPV85 not being detected. The amplicon sequence for each HPV genotype was matched completely with the original sequence obtained from the GenBank database (data not shown).

### 3.2. Relationships among Detected HPV Genotypes, Codetection Rate, and CIN Grade

**Figure 1:**
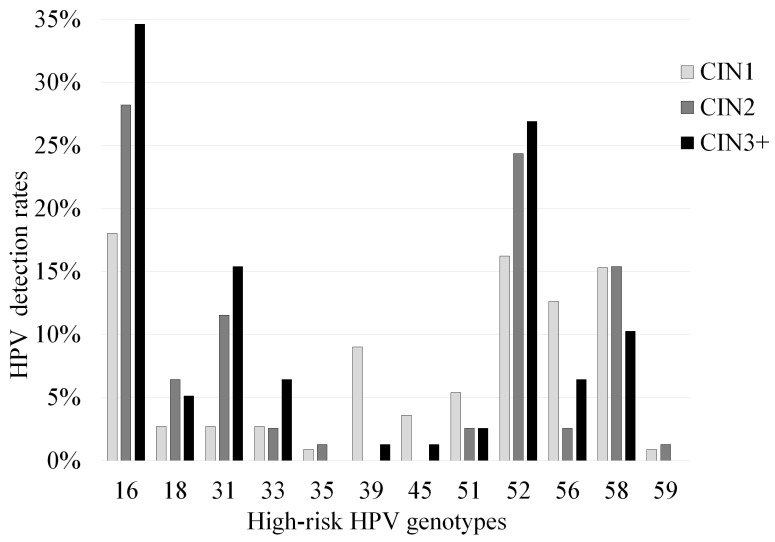
Correlation between positivity of various high-risk human papillomavirus (HPV) genotypes and cervical intraepithelial neoplasia (CIN) grade. HPV positivity includes the detection of both single and multiple high-risk HPV genotypes.

**Figure 2:**
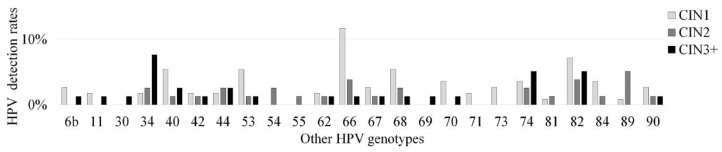
Correlation between positivity of various other human papillomavirus (HPV) genotypes and cervical intraepithelial neoplasia (CIN) grade. HPV positivity includes the detection of both single and multiple other HPV genotypes.

We summarized the relationships between detected HPV genotypes, codetection rates, and CIN grade. The correlation between the distribution of high-risk and other HPV genotypes and CIN grade was analyzed (Figure 1 and Figure 2). Genotypes such as HPV16, 52, and 58 were frequently detected in CIN1+ lesions. In CIN1 cases, HPV16 was the most frequently detected genotype, in 20 of the 87 high-risk type-positive patients (23.0%), followed by HPV52 (20.7%), HPV58 (19.5%), HPV56 (16.1%), HPV39 (11.5%), HPV51 (6.9%), HPV18, HPV31 and HPV33 (3.4% each). HPV16 was also the most frequently detected genotype in CIN2 and worse than CIN2 (CIN2+) cases (in 49 of 141 high-risk type-positive patients, 34.8%), followed by HPV52 (28.4%), HPV31 (14.9%), HPV58 (14.2%), and HPV18 (6.4%). Among these, residual analysis based on results of Chi-squared test revealed a significantly higher in the frequency of HPV16 (34.6%, 27/78), HPV31 (15.4%, 12/78), and HPV34 (7.7%, 6/78) in CIN3 cases than in CIN1 and CIN2 cases (*p* < 0.05). In contrast, HPV39, HPV56, and HPV66 were at significantly higher frequency in CIN1 than CIN2 and CIN3 (*p* < 0.01, *p* < 0.05, and *p* < 0.01).

Next, a single high-risk HPV genotype was detected in 69.7% (186/267) of cases, whereas a single other genotype was detected in 11.6% (31/267) of cases (Table 1). HPV16 was the most commonly detected single high-risk HPV type (Table 2). Codetection of high-risk HPV strains occurred in 4.9% (13/267) of cases. Codetection of high-risk plus other genotypes occurred in 22.8% (60/267) of cases. In addition, codetection of other genotypes occurred in 3.0% (8/267) of all cases. In some CIN1+ lesions with other genotypes, both single and multiple HPV genotypes were detected. No relationships were identified regarding frequency of HPV detection and CIN grade.

## 4. Discussions

In the present study, 37 of a possible 39 HPV genotypes were detected in 267 CIN cases, although notably the test did not cover all HPV types. HPV was detected in all present cases; however, HPV codetection may not be associated with CIN grade. The high-risk genotype HPV16 was the most frequently detected genotype in CIN1+ cases, while HPV34 (not a high-risk type) was significantly detected in CIN3. In general, HPV detection by the PCR method may be performed liquid-based cytology [24]. However, all scraping cells are targets for the HPV detection/diagnosis. Thus, it may not be reflected only the lesion. Accordingly, to assess HPV genotypes in the lesions, we used FFPE tissues. These results suggest that various HPV genotypes are associated with CIN across all present cases.

Most previous reports have focused on correlations between high-risk HPV infection and cervical lesions [17,18]. For example, Xu et al. [16] attempted to detect 21 HPV genotypes in 1160 CIN cases, detecting HPV in approximately 80% of cases. In another report, Evans et al. [25] observed 13 HPV genotypes, detecting HPV in approximately 90% of cases. However, over 100 genotypes of HPV have been detected in humans, and it still remains unknown exactly how many genotypes are associated with CIN [26]. Therefore, in the present study, our aim was to comprehensively detect HPV in CIN cases; we were able to detect HPV in all cases. These results indicate that the detection method used in the present study (uniplex E6/E7 PCR) may contribute to improved HPV detection rates in CIN. This may lead to a better understanding of the correlation between HPV infection and CIN.

In the study by Xiao et al. [27], a greater codetection of HPV genotypes occurred in the CIN2 group than the CIN1 group. In addition, they reported that as the grade of cervical lesion increased, the prevalence of codetection of high-risk HPV gradually decreased. Conversely, other reports have suggested that detection of a single high-risk HPV genotype, such as HPV16, was associated with CIN score [28,29]. In another report of 8977 ICC cases, HPV16, 18, 31, 33, 35, 45, 52, and 58 were the most commonly detected genotypes [30], with HPV types 16 and 18 detected in 71% of cases. Results from our study also suggest that high-risk HPV genotypes such as HPV16 are relatively frequently detected. To date, several studies have reported the relationship between codetection and CIN score, but their results are not consistent [26]. In the present study, no relationships were observed between codetection and CIN score, even in cases of codetection with high-risk HPV genotypes. These results suggest that there may not be any association between coinfection with high-risk HPV and CIN progression. However, further and larger studies are necessary to clarify these findings.

In the present study, HPV34 was shown to be significantly detected in CIN3+ cases (*p* < 0.05). HPV34 is classified into the alpha-papillomavirus group 11, a group which may be highly oncogenic, although it is not as high risk as HPV16 [31]. Jacobs et al. [32] attempted to detect HPV genotypes in 3300 liquid-based cytology cases, detecting HPV34 in 3 of 152 HPV-positive cases. However, the exact HPV mechanism associated with HPV34 is not yet fully known. In addition, HPV34 is rarely detected and little has been discussed regarding its role in the progression of CIN grade. Previous reports have also suggested that this genotype was detected in ICC [31]. In the present study, HPV34 was detected in a relatively small number of CIN3 cases. Thus, our results are compatible with earlier reports [31]. To the best of our knowledge, this may represent a new finding in Japan.

The present study has certain limitations. We detected HPV genotypes from CIN+ samples using uniplex E6/E7 PCR, which is able to identify 39 HPV genotypes. However, the present study has not demonstrated any correlation between probable high-risk HPV and CIN grade. It is difficult to clearly define chronological changes in CIN because of the need for histology and HPV genotyping in the short-term. As another limitation, a limited number of cases were included in this study, with a smaller number of CIN2 and CIN3 cases compared to CIN1. However, although the number of cases was limited, HPV was detected in all CIN cases in this study. Therefore, extensive HPV typing is important to clarify the relationship between genotype, superinfection, and CIN. In order to investigate the progression of CIN to cancer, it is necessary to include a greater number of cases and analyze CIN2+ cases in greater detail. Moreover, we did not introduce the primary HPV screening in this study [33]. However, to establish a model for reconsidering the genotype, this screening should be introduced in Japan in the near future [33].

In conclusion, we demonstrated by a classical PCR assay that all CINs are caused by HPV with equal sensitivity using a type-specific primer targeting the E6 or E7 genes. To precisely assess the correlation between HPV infection profiles and cervical lesions, including CIN and ICC, the comprehensive detection of HPV genomes from lesions using such sensitive methods is essential. Since the number of cases is small in this study, further and larger studies are necessary to target 13 HPV genotypes, including HPV34, when introducing primary screening for cervical cancer by HPV testing in Japan.

## Figures and Tables

**Table 1:** Relationships among detected human papillomavirus (HPV) genotypes, codetections, and cervical intraepithelial neoplasia (CIN).

	Single HPV Genotype Detected			Multiple HPV Genotypes Detected			
	High-Risk	Other	Total	High-Risk	High-Risk + Other	Other	Total
	Genotypes	Genotypes		Genotypes	Genotypes	Genotypes	
CIN1	47.70%	18.00%	65.80%	3.60%	27.00%	3.60%	34.20%
N = 111	(53/111)	(20/111)	(73/111)	(4/111)	(30/111)	(4/111)	(38/111)
CIN2	66.70%	6.40%	73.10%	5.10%	17.90%	3.80%	26.90%
N = 78	(52/78)	(5/78)	(57/78)	(4/78)	(14/78)	(3/78)	(21/78)
CIN3	64.10%	7.70%	71.80%	6.40%	20.50%	1.30%	28.20%
N = 78	(50/78)	(6/78)	(56/78)	(5/78)	(16/78)	(1/78)	(22/78)
Total	58.10%	11.60%	69.70%	4.90%	22.50%	3.00%	30.30%
N = 267	(155/267)	(31/267)	(186/267)	(13/267)	(60/267)	(8/267)	(81/267)

**Table 2:** Detection rate of each human papillomavirus (HPV) genotype in cervical intraepithelial neoplasia (CIN).

High-Risk Genotype	Single Detection	Multiple Detection (High-Risk Genotypes Alone)	Multiple Detection (High-Risk and Other Genotypes)	Total
16	78.3% (54/69)	5.8% (4/69)	15.9% (11/69)	26.8% (69/257)
18	75.0% (9/12)	16.7% (2/12)	8.3% (1/12)	4.7% (12/257)
31	58.3% (14/24)	16.7% (4/24)	25.0% (6/24)	9.3% (24/257)
33	50.0% (5/10)	0.0% (0/10)	50.0% (5/10)	3.9% (10/257)
35	0.0% (0/2)	0.0% (0/2)	100% (2/2)	0.8% (2/257)
39	45.5% (5/11)	9.1% (1/11)	45.5% (5/11)	4.3% (11/257)
45	75.0% (3/4)	0.0% (0/4)	25.0% (1/4)	1.6% (4/257)
51	22.2% (2/9)	0.0% (0/9)	77.8% (7/9)	3.5% (9/257)
52	51.8% (29/56)	14.3% (8/56)	33.9% (19/56)	21.8% (56/257)
56	38.1% (8/21)	14.3% (3/21)	47.6% (10/21)	8.2% (21/257)
58	70.3% (26/37)	10.8% (4/37)	18.9% (7/37)	14.4% (37/257)
59	0.0% (0/2)	50.0% (1/2)	50.0% (1/2)	0.8% (2/257)
Total	60.3% (155/257)	10.5% (27/257)	29.2% (75/257)	
